# Conservation in the face of climate change: recent developments

**DOI:** 10.12688/f1000research.6490.1

**Published:** 2015-10-28

**Authors:** Joshua Lawler, James Watson, Edward Game

**Affiliations:** 1 School of Environmental and Forest Sciences, University of Washington, Seattle, WA, USA; 2 School of Geography, Planning, and Environmental Management, University of Queensland, St. Lucia, Queensland, Australia; 3 Wildlife Conservation Society, Global Conservation Program, Bronx, NY, USA; 4 The Nature Conservancy, West End, Queensland, Australia; 5 School of Biological Sciences, University of Queensland, St. Lucia, Queensland, Australia

**Keywords:** climate change, conservation, restoration, assisted colonization, biodiversity, extinction

## Abstract

An increased understanding of the current and potential future impacts of climate change has significantly influenced conservation in practice in recent years. Climate change has necessitated a shift toward longer planning time horizons, moving baselines, and evolving conservation goals and targets. This shift has resulted in new perspectives on, and changes in, the basic approaches practitioners use to conserve biodiversity. Restoration, spatial planning and reserve selection, connectivity modelling, extinction risk assessment, and species translocations have all been reimagined in the face of climate change. Restoration is being conducted with a new acceptance of uncertainty and an understanding that goals will need to shift through time. New conservation targets, such as geophysical settings and climatic refugia, are being incorporated into conservation plans. Risk assessments have begun to consider the potentially synergistic impacts of climate change and other threats. Assisted colonization has gained acceptance in recent years as a viable and necessary conservation tool. This evolution has paralleled a larger trend in conservation—a shift toward conservation actions that benefit both people and nature. As we look forward, it is clear that more change is on the horizon. To protect biodiversity and essential ecosystem services, conservation will need to anticipate the human response to climate change and to focus not only on resistance and resilience but on transitions to new states and new ecosystems.

## Introduction

Climate change is one of the largest threats to biodiversity and to natural systems in general ^1, 2^. Recent changes in climate are driving shifts in the timing of ecological events ^3^, the distribution of species ^4, 5^, and the functioning of ecosystems ^6^. Models project even greater changes for the future ^7– 10^.

These climate-driven changes challenge the way the planning and practice of conservation have traditionally been done. Most conservation has been predicated on the fact that the environment is relatively stable over the time frames of management planning (generally less than 50 years). However, projected changes in species distributions and ecosystem functions present obvious challenges to this assumption. Over the last decade, there has been a growing literature on what climate change means for biodiversity ^11^ and the implications this has for conservation planning and action ^12^. As a consequence, there have been some notable shifts in the way conservation is being conducted, from the goals and structure of conservation organizations to the planning and execution of conservation projects on the ground ^12, 13^.

Climate change is increasingly integrated into the daily operations of conservation organizations. The anticipated impacts of climate change have driven planners and managers to consider longer time horizons and to anticipate the potentially synergistic effects of climate change and other threats and the need to address them quickly. Likewise, conservation biologists have begun to acknowledge the importance of planning for extreme weather events in addition to slow, long-term climatic change. These shifts have led to new perspectives on, and alterations to, approaches used to conserve biodiversity. Here, we describe some of the more prominent changes in the approaches conservation planners and practitioners have taken to help address the threat of climate change. Some of these approaches, such as restoration and the prioritization of species for conservation actions, have been around for quite some time but needed reframing as a consequence of the likely impacts of climate change. Others, such as assisted colonization, are new twists on old practices and have arisen directly out of the challenges posed by climate change.

## Changing conservation approaches to address climate change

### Rethinking restoration

Restoration is one of the basic tools of a conservation practitioner. It has traditionally involved returning a system to its state prior to some disturbance ^14^. However, climate change challenges the very nature of restoration based on such a definition ^15^. It brings into question the utility of historical benchmarks as restoration targets, the species or seed sources to be used, and the time frame for planning.

Rethinking how restoration efforts are applied in light of climate change has led to shifts in thinking as well as in practice. Most fundamentally, practicing restoration in a changing climate requires embracing uncertainty and accepting that the goals of a project may need to change over time ^16^. Instead of relying on historical benchmarks, restoration efforts will likely need to look into the future and anticipate change—perhaps relying on a dynamic reference process that accounts for variability in reference ecosystems ^16, 17^. Looking forward will also include rethinking the mix of species to be planted and potentially focusing on ecosystem function rather than particular assemblages of species. Restoration efforts have begun to make use of the same niche modelling methods that have been used to assess potential species responses to climate change ^18^, but there is clear recognition that better models are needed ^19^.

Many recent climate-adaptation efforts have involved restoration. Of the projects funded by the Wildlife Conservation Society’s Climate Adaptation Fund, at least 70% have involved restoration ^20^. These projects have included riparian restoration to enhance connectivity, coastal restoration to prevent storm damage, forest restoration to reduce fire risk, and prairie restorations to enhance watershed function. In addition to the projects that clearly engage in restoration, several projects are aimed at converting one type of ecosystem to another—not necessarily restoring a historical condition but preparing an ecosystem to function differently in a future climate. An example of this type of project involved converting forested areas to grasslands to facilitate marsh establishment in the face of sea-level rise. Whether such actions are classified as restoration could be debated, but clearly the lessons learned from decades of restoration will be essential to these new types of adaptation projects.

### Changes in how conservation planning is undertaken

Systematic conservation planning ^21^ has been used widely around the world to help prioritize conservation efforts, particularly the location of protected areas ^22^. Most conservation planning has been based on “static” representations of biodiversity across a region, an approach that is clearly challenged by climate-driven changes in the distribution of species and communities ^23^. Consequently, substantial thought has been put into how best to incorporate climate change into the conservation-planning process.

The cornerstone of initial approaches to integrate climate change into conservation planning was the use of correlative niche models to predict future distributions of species and ensure that these were adequately represented by present-day conservation efforts. The more sophisticated niche-based planning efforts included the ability of species to track changing habitat conditions through space and time ^24, 25^ and consideration of uncertainties in predicted distributions ^26, 27^. Climate, however, is only one of many factors determining the distributions of species, and the relationship is complex, uncertain, and in many cases evolving ^28^. The magnitude of these ecological uncertainties compounded by the uncertainties associated with climate predictions ^29^ led to calls to integrate climate change into planning using approaches that were more robust to uncertainty in predicted climate impacts ^30– 32^.

An approach to planning that has gained some traction for conservation in a dynamic climate involves conserving the underlying geophysical variation in a region, also referred to as “conserving nature’s stage” ^33^. The rationale for this approach is that, theoretically, there should be a strong relationship between species distributions and geophysical settings (for example, elevation and geology) ^34^ such that conserving representative examples of geophysical settings will protect representative ecological communities under both current and future climates ^35^. A similar approach that focuses on current and known patterns in a region emphasizes conserving connectivity between climatically diverse areas ^31, 36, 37^.

### Increasing connectivity

Increasing the connectivity of landscapes to allow species to move in response to climate change is the most-often cited climate change adaption strategy ^38– 40^. Traditionally, connectivity planning has focused on connecting patches of habitat with what amount to linear strips or stepping-stones of more habitat. Although this approach may allow species to move through the landscape, it may do little to facilitate movement into what might become newly suitable habitat. Early attempts to model connectivity expressly for addressing climate change involved projecting shifts in species distributions through time and either identifying overlap of current and future distributions or mapping pathways that tracked those shifting distributions ^41, 42^. A more mechanistic application of this approach involved mapping potential corridors through climate-driven shifts in suitable levels of snowpack for wolverines in the northwestern United States ^43^. Other studies have taken different approaches to identifying important areas for species movement in the face of climate change—approaches that are less reliant on projected future changes in climate and species distributions. Brost and Beier ^44^ and Beier ^45^ focused on the geophysical settings mentioned in the previous section, and charted routes across the landscape that either connected similar geophysical setting or linked a diversity of settings. Nuñez and colleagues ^46^ mapped routes through landscapes that connected slightly warmer patches of intact land to slightly cooler ones with routes that followed gentle temperature gradients and avoided human-impacted landscapes.

Although efforts continue to develop more relevant climate-connectivity methods, the critical question of whether corridors are really needed—or whether there might be other strategies and approaches that would achieve the same result—has been repeatedly raised ^37^. Some argue that the focus on corridors is misguided and that, alternatively, protecting large intact ecosystems should be prioritized ^47, 48^. Others have argued that the benefits of increasing the size and number of corridors are fewer than those resulting from simply increasing the amount of protected land, which, if regularly distributed, would increase connectivity ^49^.

### Assessing extinction risk through the climate change lens

Although a growing wealth of studies predict increased extinction risk for species because of climate change ^50, 51^, many of these vary enormously in their estimations. It is also increasingly recognized that the predictions of extinction risk do not reflect the number of species that have become vulnerable (or extinct) to date, nor do they match the number identified as threatened because of climate change on the International Union for the Conservation of Nature (IUCN) Red List (only 10.5% of the 22,176 species) ^52^. A fundamental challenge has involved integrating the projections of species niche models (the most-often used tool for assessing the impacts of climate change on species) into the processes of real-world extinction assessments.

Simple measures of population size, geographic range size, and other indicators of current status already used as IUCN Red List criteria are likely to be good predictors of climate change-associated extinction risk ^53^. Such IUCN Red List criteria have been used to predict the risk of extinction in the absence of conservation action and the time lag between assessment and extinction ^54, 55^. This time lag amounts to a warning period in which adaptation efforts can be taken to prevent extinction. Although there is a warning period, it is finite and thus delays in developing and implementing conservation plans after a species is identified as being threatened could be costly. Roughly half of listed species are likely to go extinct within 20 years of being listed as critically endangered ^54^.

An additional challenge is that most species risk assessments treat climate change as a problem driven by relatively slow, predictable, and continuous change in environmental conditions and fail to account for other important components of climate change, such as increasing extreme weather and climate events (for example, cyclones, floods, and drought) ^56, 57^. It is increasingly recognized that the increases in frequencies and intensities of extreme events are critical determinants of patterns of biological diversity and will affect it differently from impacts resulting from steady climate change ^58^. A good example of this is how climate change will impact bat species: extreme maximum temperature is now considered a critical factor in the vulnerability of bats to climate change ^59^, but many studies (for example, 60) fail to consider it in projections of species distributions under climate change.

Even though our understanding of which extremes are most important and how they are shifting is limited ^61^, there are good examples of assessments that do account for extremes. Recently, Ameca y Juárez and colleagues ^62^ produced a comprehensive analysis of the impacts of cyclones and droughts on terrestrial mammals, one of the few large-scale studies to consider exposure to extreme events. They followed this exposure analysis with an assessment of terrestrial mammal sensitivity to extreme weather and climate events, identifying biological traits that make large terrestrial mammals more susceptible to climate-induced population declines.

Alongside species risk assessments, the assessment of the vulnerability of species to climate change—as well as the climate-related vulnerability of places and natural resources in general—has emerged as an important step in the adaptation process ^63^. As with extinction risk assessments, many different approaches to assessing vulnerability have been proposed (for example, 64), each evaluating some subset or combination of sensitivity, exposure, and adaptive capacity ^65^. These vulnerability assessments serve not only to determine which species are likely to be most vulnerable but also to identify the factors that make a species vulnerable and thus potential conservation actions—adaptation measures—that can be taken to reduce vulnerability.

### Assisted colonization

One of the relatively new tools in the conservation toolbox is assisted colonization—broadly defined by the IUCN as the movement of an organism outside of its native range to avoid extinction of populations due to current or future threats ^66^. There are some species, particularly endemics with relatively specific habitat requirements and poor dispersal abilities, that will be unable to move to suitable climates. When these species are threatened with extinction—because of either climate change or some other factor—it may become necessary to move them to prevent their loss.

The question of whether assisted colonization should even be considered as an option spawned a lively debate ^67– 72^. Those opposed to assisted colonization argue that the history of invasive species has taught us that the potential impacts on the ecosystems into which organisms would be moved could be too great ^68^. Those in favour of keeping the option of assisted colonization on the table argue that it would likely be necessary for preventing the extinction of certain species ^69^, that the potential impact of translocated species is likely overstated for several reasons ^69, 71^ (including that the traits of species that will need to be moved are not those traits generally associated with invasive species ^73^), and that the amount of change that systems will experience over the coming decades will likely overshadow the impacts of translocated individuals of a rare and declining species ^74^.

As the debate worked its ways through the scientific literature, many researchers started to ask more productive questions—facing the reality that assisted colonization was already being used. There were, for example, several early efforts to develop frameworks for determining under what circumstances assisted colonization would be a viable conservation option ^75, 76^. Other studies have highlighted the importance of the timing of assisted colonization efforts ^77^, developed advanced modelling approaches for identifying potential sites for translocations ^78^, and explored the situations in which invasions will be less likely and hence assisted colonization a less risky venture ^79^. Furthermore, calls for the development of policies to address assisted colonization ^80^ have begun to be met ^81, 82^. Overall, it appears that, at least in the scientific literature, assisted colonization is gaining acceptance as a tool in the conservation toolbox and one that may not differ so much from other movements of species for conservation reasons ^83, 84^.

## Future trends

The practice, and to some degree the study, of conservation is currently undergoing a major shift—a shift from a focus on nature to a focus on nature and people. The idea that people are a part of ecosystems and that conservation needs to include the social sciences is not new and this is not the shift to which we are referring. This new shift is one from conserving nature for nature’s sake to conserving nature both for nature’s sake and for the use and enjoyment by people ^85^. This shift has resulted in an apparent change in the missions and the actions of several major non-governmental conservation organizations (for example, The Nature Conservancy, the World Wildlife Fund, and Conservation International). Like the subject of assisted colonization, however, this shift has not been well received by all in the conservation community and there remains a heated debate in conservation circles as well as in the literature about the degree to which conservation should focus on the needs of people ^85– 88^.

The impact of this shift can be clearly seen in the way that conservation organizations are addressing climate change and is reflected in the application of all of the approaches mentioned above. With respect to restoration, conservation planning, and connectivity, conservation practitioners have begun to target efforts that consider the roles that natural systems play in protecting people against the potential impacts of climate change ^89, 90^. These ecosystem-based adaptation strategies may be more cost-effective than hard infrastructure-based solutions. One striking example of this approach is The Nature Conservancy’s “flood plains by design” strategy in which stretches of river are restored in places that will simultaneously reduce flooding of nearby communities and restore fish habitat. Another example is the active protection of coastal habitats, which has the potential to reduce the risks and the costs of sea-level rise, providing a critical service in the face of climate change ^91^.

Because climate-driven changes are likely to be so large in some places, climate change is in part causing conservation practitioners to question their goals as well as the approaches they use ^7^. These new goals are beginning to take people’s needs into account. For example, restoration efforts are now being refocused toward ecosystem function and ecosystem services instead of the specific set of species in a given ecosystem ^19^. In addition, assisted colonization may be called on not just to preserve threatened species, but also to provide certain functions—and perhaps to allow ecosystems to provide certain services ^73, 92^.

Given that our understanding of climate change impacts is still evolving, the theory and practice of conservation will likely continue to change at a relatively fast pace. One of the greatest future challenges to the conservation of biodiversity will likely come from how people respond to climate change ^93^. Sea-level rise is forcing human populations to consider radical adaption action, including the construction of massive sea walls and the migration of coastal and island communities ^94, 95^. Water shortages and crop failures will similarly result in human migrations, shifts in agriculture, and increased water withdrawals. There is increasing recognition that, in many places, human responses to climate change may further constrain options for biodiversity conservation, and therefore planning needs to simultaneously consider both human and biodiversity responses ^93^. The tools that conservation practitioners have to address climate change (for example, conservation planning, restoration, species risk assessments, and assisted colonization) will likely be most effective if their application takes human responses to climate change into account.

Continued rapid climate change will also necessitate a shift from discussions of resistance and resilience to more strategies that embrace change and foster transitions ^96^. Particularly if society hopes to continue to be the recipient of essential ecosystem services and to enjoy a diversity of plants and animals, conservation efforts will need to focus on smoothly transitioning ecosystems from one state to another. The enormity of that challenge necessitates policies and actions that reduce greenhouse-gas emissions and increase carbon sequestration. Unless adaptation is accompanied by meaningful mitigation efforts, it will be hard for conservation practitioners to accomplish even their shifting and evolving goals.

## Abbreviation

IUCN, International Union for the Conservation of Nature.
